# An animal model of differential genetic risk for methamphetamine intake

**DOI:** 10.3389/fnins.2015.00327

**Published:** 2015-09-23

**Authors:** Tamara J. Phillips, Shkelzen Shabani

**Affiliations:** ^1^VA Portland Health Care SystemPortland, OR, USA; ^2^Department of Behavioral Neuroscience and Methamphetamine Abuse Research Center, Oregon Health & Science UniversityPortland, OR, USA; ^3^Department of Biology, Minot State UniversityMinot, ND, USA

**Keywords:** addiction, amphetamine, aversion, drinking, reward, hypothermia, hyperthermia, TAAR1

## Abstract

The question of whether genetic factors contribute to risk for methamphetamine (MA) use and dependence has not been intensively investigated. Compared to human populations, genetic animal models offer the advantages of control over genetic family history and drug exposure. Using selective breeding, we created lines of mice that differ in genetic risk for voluntary MA intake and identified the chromosomal addresses of contributory genes. A quantitative trait locus was identified on chromosome 10 that accounts for more than 50% of the genetic variance in MA intake in the selected mouse lines. In addition, behavioral and physiological screening identified differences corresponding with risk for MA intake that have generated hypotheses that are testable in humans. Heightened sensitivity to aversive and certain physiological effects of MA, such as MA-induced reduction in body temperature, are hallmarks of mice bred for low MA intake. Furthermore, unlike MA-avoiding mice, MA-preferring mice are sensitive to rewarding and reinforcing MA effects, and to MA-induced increases in brain extracellular dopamine levels. Gene expression analyses implicate the importance of a network enriched in transcription factor genes, some of which regulate the mu opioid receptor gene, *Oprm1*, in risk for MA use. Neuroimmune factors appear to play a role in differential response to MA between the mice bred for high and low intake. In addition, chromosome 10 candidate gene studies provide strong support for a trace amine-associated receptor 1 gene, *Taar1*, polymorphism in risk for MA intake. MA is a trace amine-associated receptor 1 (TAAR1) agonist, and a non-functional *Taar1* allele segregates with high MA consumption. Thus, reduced TAAR1 function has the potential to increase risk for MA use. Overall, existing findings support the MA drinking lines as a powerful model for identifying genetic factors involved in determining risk for harmful MA use. Future directions include the development of a binge model of MA intake, examining the effect of withdrawal from chronic MA on MA intake, and studying potential *Taar1* gene × gene and gene × environment interactions. These and other studies are intended to improve our genetic model with regard to its translational value to human addiction.

## Introduction

Methamphetamine (MA) is a highly addictive and potent psychostimulant drug. The 2012 National Survey on Drug Use and Health reported that ~5% of the population (over 12 million people) has tried MA at least once. Addiction has been described as a chronic, relapsing disease that is associated with long-term neurological changes as a result of chronic drug use. Compulsive drug seeking, despite negative consequences, is a hallmark (Joffe et al., 2014). Not all individuals who try MA develop a pattern of use that can be characterized as addiction. However, for those that do, adverse physical, cognitive, and psychological effects are likely to be experienced (Herbeck et al., 2013; Panenka et al., 2013; Astarita et al., 2015). Risk for higher levels of MA use may have a genetic component that we do not yet fully understand.

The euphoric and other rewarding effects of MA are likely factors that underlie its addiction potential. In fact, MA can increase brain dopamine (DA) levels, as well as serotonin and norepinephrine levels, and thereby have an impact on brain circuitry underlying the experience of reward (Fleckenstein et al., 2007). However, some acute effects of MA are also likely to be experienced as aversive. For example, MA can increase heart rate and blood pressure, and can activate the “stress axis,” which is involved in the flight or fight reaction to stressors and in maintaining homeostasis (Polesskaya et al., 2011; Rusyniak et al., 2012; Zuloaga et al., 2015). Sensitivity to rewarding effects of MA that could increase risk for continued MA use may be countered, in some individuals, by sensitivity to physiological effects that are experienced as aversive. Thus, examination of both types of MA effects, and their balance (Cruickshank and Dyer, 2009; Davis and Riley, 2010), is important in identifying individuals who are at greatest risk for MA addiction. Furthermore, identification of genetic factors that influence sensitivity to these effects would suggest important prevention and treatment targets.

## Genetic factors and risk for MA addiction

### Human data

Data from family, twin and adoption studies overwhelmingly indicate that risk for substance abuse has a heritable component (Kendler et al., 2003; Ystrom et al., 2014). Environment is also important (McGue et al., 2000); however, in an analysis of six classes of illicit substances, shared environment had a greater impact on use than on abuse/dependence (Kendler et al., 2003). Furthermore, although parent-offspring transmission involves both environmental and genetic influences, genetic factors appear to have a stronger role (Kendler et al., 2015), and some findings indicate that the heritable influence is not substance-specific (Kendler et al., 2003, 2015; Uhl et al., 2008). A number of candidate genes have been nominated for further study for their potential association with amphetamine (AMPH) sensitivity (Hart et al., 2012) or a MA use disorder (Aoyama et al., 2006; Bousman et al., 2009), though none have yet been confirmed. Far less research has been done in this area for AMPHs than for some other addictive substances, such as alcohol and nicotine (e.g., Enoch, 2013; Loukola et al., 2014; Samochowiec et al., 2014; Wen et al., 2014; Bühler et al., 2015). One logical reason for this is that MA abuse has existed for less time, and another is that the population size for MA abuse is smaller, making it difficult to obtain adequate sample sizes for genome-wide association studies.

### Animal data

In 2008, we reviewed the existing behavioral genetics literature on addiction-related AMPH and MA effects (Phillips et al., 2008). We found that the relevant literature consisted of a large number of studies using single gene knockout mice and that traits related to sensitivity to various effects of AMPHs were well-represented, but the literature was lacking in the area of reward- and consumption-related traits. Since then, a few studies in this area have emerged. For example, genetic deletion of two types of melatonin receptors was found to eliminate MA-induced conditioned place preference (CPP), suggesting a role in MA-related reward (Clough et al., 2014). Mice lacking the neurotrophic factor, pleiotrophin, developed normal AMPH-induced CPP that was sustained for a longer period of time than in their WT counterparts (Gramage et al., 2010; Martin et al., 2013). Increased expression of glial cell line-derived neurotrophic factor in the striatum of mice reduced intravenous self-administration of MA and cue-induced reinstatement of MA seeking behavior (Yan et al., 2013). Some recent papers have also reported genome-wide profiling of MA-induced effects. An advanced intercross mouse line was used in a genetic analysis of sensitivity to the acute stimulant effects of MA (Parker et al., 2012) and 7 genome-wide significant quantitative trait loci (QTL) were mapped (Parker et al., 2012). Cadet et al. (2013) identified MA-regulated genes in the rat striatum and documented MA effects on histone acetylation. Mulligan et al. (2013) focused on the regulation of micro-RNA biogenesis genes in the brain across multiple drugs of abuse including MA, and found roles in several behavioral phenotypes. However, none of these genome-wide analyses have been for MA-consumption related traits, for which genetic mouse models have been lacking, whereas such models are plentiful for alcohol (e.g., Morozova et al., 2012; Barr, 2013; Becker, 2013; Crabbe, 2014a,b). To address this deficiency, we took the lead from models created for alcohol consumption and created an animal model of genetically-determined differences in voluntary MA intake. Tables 1, 2 summarizes our published findings so far.

**Table 1:** **Published MA traits characterized in the MA drinking lines**.

**Trait**	**Drug dose**	**Results**	**References**
MA drinking; 2-bc 18-h	20, 40 mg/l	MAHDR-1 > MALDR-1	Wheeler et al., 2009
MA drinking; 2-bc 18-h	20, 40 mg/l	MAHDR-2 > MALDR-2	Shabani et al., 2011
MA drinking; 2-bc 18-h	20, 40 mg/l	MAHDR-3 > MALDR-3	Harkness et al., 2015
MA drinking; 2-bc 4-h; lickometer	20, 40, 80 mg/l	MAHDR-2 > MALDR-2	Eastwood et al., 2014
MA blood levels after 2-bc 4-h	20, 40 mg/l	MAHDR-2 > MALDR-2	Eastwood et al., 2014
Operant ICV MA self-administration	0.1–2.5 ug/infusion	MAHDR-1 > MALDR-1	Shabani et al., 2012a
Operant oral MA self-administration	20, 40 mg/l	MAHDR-2 > MALDR-2	Shabani et al., 2012a
MA-induced CPP: Drug-free CPP test	0.5 mg/kg	Preference only in MAHDR-1	Wheeler et al., 2009
MA-induced CPP: Drug-present CPP test	0.5 mg/kg	Preference only in MAHDR-1	Wheeler et al., 2009
MA-induced CPP: Drug-free CPP test	0.5, 2, 4 mg/kg	Preference only in MAHDR-2	Shabani et al., 2011
MA-induced CPP: Drug-present CPP test	0.5, 2, 4 mg/kg	MAHDR-2 preference; MALDR-2 aversion	Shabani et al., 2011
MA-induced locomotor stimulation: acute	0.5, 2, 4 mg/kg	MAHDR-2 = MALDR-2	Shabani et al., 2011
MA-induced locomotor sensitization	0.5, 2, 4 mg/kg	MAHDR-2 > MALDR-2	Shabani et al., 2011
MA-induced CPA	2, 4 mg/kg	MAHDR-2 < MALDR-2	Shabani et al., 2012b
MA-induced CTA	1, 2 mg/kg	CTA only in MALDR-1	Wheeler et al., 2009
MA-induced CTA	1, 2, 4 mg/kg	CTA only in MALDR-2	Shabani et al., 2012b
MA-induced hyperthermia	1, 2, 4, 8, 16 mg/kg	Hyperthermia only in MAHDR-2	Harkness et al., 2015
MA-induced hyperthermia	1, 2, 4, 8, 16 mg/kg	MAHDR-3 > MALDR-3	Harkness et al., 2015
MA-induced hypothermia	1, 2, 4 mg/kg	Hypothermia only in MALDR-2	Harkness et al., 2015
MA-induced hypothermia	2, 4 mg/kg	Hypothermia only in MALDR-3	Harkness et al., 2015
MA-induced behavioral inhibition	0.5, 1, 2, 4 mg/kg	MAHDR-1 = MALDR-1	Moschak et al., 2012
MA drinking on circadian period length	25, 50 mg/l	MAHDR-2 < MALDR-2	Olsen et al., 2013
MA-induced DA release in NAc	2 mg/kg	MAHDR-2 = MALDR-2	Lominac et al., 2014
MA-induced 5-HT release in NAc	2 mg/kg	MAHDR-2 = MALDR-2	Lominac et al., 2014
MA-induced DA release in mPFC	2 mg/kg	MAHDR-2 > MALDR-2	Lominac et al., 2014
MA-induced 5-HT release in mPFC	2 mg/kg	MAHDR-2 < MALDR-2	Lominac et al., 2014

2-bc, 2-bottle choice; 5-HT, serotonin; CPP, conditioned place preference; CPA, conditioned place aversion; CTA, conditioned taste aversion; DA, dopamine; ICV, intracerebroventricular; MA, methamphetamine; MAHDR, methamphetamine high drinking; MALDR, methamphetamine low drinking; mPFC, medial prefrontal cortex; NAc, nucleus accumbens.

**Table 2:** **Published non-MA traits characterized in the MA drinking lines**.

**Trait**	**Drug dose**	**Results**	**References**
Quinine drinking; 2-bc 24-h	0.015, 0.03 mM	MAHDR-1 = MALDR-1	Wheeler et al., 2009
Quinine drinking; 2-bc 24-h	0.015, 0.03 mM	MAHDR-2 = MALDR-2	Shabani et al., 2011
Saccharin drinking; 2-bc 24-h	0.033, 0.066%	MAHDR-1 = MALDR-1	Wheeler et al., 2009
Saccharin drinking; 2-bc 24-h	0.033, 0.066%	MAHDR-2 = MALDR-2	Shabani et al., 2011
KCl drinking; 2-bc 24-h	100, 200 mM	MAHDR-1 = MALDR-1	Wheeler et al., 2009
KCl drinking; 2-bc 24-h	100, 200 mM	MAHDR-2 = MALDR-2	Shabani et al., 2011
Voluntary morphine intake 2-bc 24-h	0.3, 0.7, 1 mg/ml	MAHDR-2 < MALDR-2	Eastwood and Phillips, 2014
Basal locomotor activity	saline	MAHDR-1 = MALDR-1	Wheeler et al., 2009
Basal locomotor activity	saline	MAHDR-2 = MALDR-2	Shabani et al., 2011
COC-induced CPP: Drug-free CPP test	10 mg/kg	MAHDR-2 = MALDR-2	Gubner et al., 2013
COC-induced locomotor stimulation: acute	5, 10, 20, 30 mg/kg	MAHDR-2 = MALDR-2	Gubner et al., 2013
COC-induced CTA	15, 30 mg/kg	MAHDR-2 = MALDR-2	Gubner et al., 2013
Ethanol-induced hypothermia	2, 4 g/kg	MAHDR-2 = MALDR-2	Harkness et al., 2015
Novel object recognition	naive	MAHDR-2 = MALDR-2	Olsen et al., 2013
Spatial memory retention	naive	MAHDR-2 < MALDR-2	Olsen et al., 2013
Fear conditioning	naive	MAHDR-2 = MALDR-2	Olsen et al., 2013
Fentanyl analgesic effects	0.05, 0.1, 0.2, 0.4 mg/kg	MAHDR-2 = MALDR-2	Eastwood and Phillips, 2012
Basal locomotor activity	saline	MAHDR-2 < MALDR-2	Eastwood and Phillips, 2012
Fentanyl locomotor effects	0.1, 0.2, 0.4 mg/kg	MAHDR-2 < MALDR-2	Eastwood and Phillips, 2012
Morphine locomotor effects	10, 20, 30 mg/kg	MAHDR-2 < MALDR-2	Eastwood and Phillips, 2012

2-bc, 2-bottle choice; COC, cocaine; CPP, conditioned place preference; CTA, conditioned taste aversion; KCl, potassium chloride; MA, methamphetamine; MAHDR, methamphetamine high drinking; MALDR, methamphetamine low drinking.

## Development of an animal model of genetic risk for MA intake

Intravenous operant self-administration has been considered the gold standard for modeling human drug use in rodents, because several features of addiction can be assessed, including acquisition of drug use, strength of motivation, and relapse-like behavior in response to drug-associated cues, stressors and the drug itself (Nawata et al., 2012; Ma et al., 2013; Sharpe et al., 2015). However, this method is not conducive to testing the number of animals required for a selective breeding project (~600 animals for a short-term selective breeding project; 120 animals per generation). We created selected lines for high and low MA intake using a voluntary drinking procedure (Wheeler et al., 2009; Shabani et al., 2011). Justification for the approach can be found in our previous publications and model validation studies are described below. Relevant to the model is that humans administer MA via the oral route, MA is readily absorbed via the digestive tract, and its use via this route can lead to dependence that is similar to that seen when it is administered intravenously or via the nasal route (Sulzer et al., 2005; United Nations Office on Drugs and Crime, 2010).

The F2 cross of the C57BL/6J (B6) and DBA/2J (D2) inbred mouse strains were the originating population of mice used to develop the MA high drinking (MAHDR) and MA low drinking (MALDR) lines. This cross was chosen to allow comparison of our genetic results to those for MA and other drug traits examined previously in B6 and D2 mice or in other genetic models derived from them, such as recombinant inbred strains (Grisel et al., 1997; Phillips et al., 1998; Bergeson et al., 2001; Janowsky et al., 2001; Kirstein et al., 2002; Fehr et al., 2005; Palmer et al., 2006; DuBose et al., 2013). Two-bottle choice MA drinking was measured in 120 F2 mice. The mice were individually housed and allowed during a 48-h period to consume fluid from 25-ml graduated cylinders fitted with stoppers and ball-bearing sipper tubes. For the next 4 days, they were offered a tube containing tap water and another containing 20 mg of MA per liter of tap water (20 mg/l) for an 18-h period, beginning 3 h before the dark cycle began. On the subsequent 4 days, the MA concentration offered was increased to 40 mg/l. The relative position of the MA tube to the water tube was alternated every 2 days. During the additional 6 h of each day, the mice had free access to a single tube containing tap water. Food was available at all times. Drinking volumes for all tubes were measured at the beginning and end of the 18-h period and also for the 6-h water-only periods. These data were used to calculate mg of MA consumed per kg of body weight and preference for the MA-containing solution, which was calculated by dividing the volume (ml at an accuracy of 0.2 ml) consumed from the MA tube by total volume consumed from the water and MA tubes during the 18-h period. To identify the animals that consumed the most and the least MA, average amount consumed in mg/kg on days 2 and 4, when the 40 mg/l concentration was offered, was used. Days 2 and 4 were second days after a MA tube position switch and use of data from these days reduces variability in volume intake associated with the animal having to locate the MA-containing tube.

The decision to offer MA for 18 vs. 24 h a day followed a colleague's advice; he suggested that anorectic-like effects were less likely to occur with sub-chronic access. However, we also wanted to provide longer periods of access to MA, to allow animals to consume relatively high amounts. Other research also indicates that longer access periods result in higher levels of MA self-administration in rats (Recinto et al., 2012). We have not yet published data comparing intake for 18 vs. 24 h a day in the MA drinking (MADR) lines, nor have we examined the impact of differing withdrawal periods, though such studies are planned.

We have completed the selective breeding of 3 sets of MAHDR and MALDR lines. Response to selection across generations has been published for two sets of these lines (Wheeler et al., 2009; Shabani et al., 2011) and results are comparable for the third set (Harkness et al., 2015). These replicate sets of lines were consecutively created, at an ~2-year interval. There is high consistency of the selective breeding results. There are several advantages to producing consecutive vs. simultaneous replicate lines. These include a smaller colony space requirement, lower level resources spread across time, and the ability to perform identical replication studies, as well as extend studies based on results in former lines. One example of such an extension is a study that we recently performed to examine pattern of MA intake over time in the replicate 2 MAHDR and MALDR lines (Eastwood et al., 2014). A lickometer system was used to determine pattern of MA drinking in addition to amount consumed. Pattern variables analyzed were latency to first bout of licks, the number of bouts, bout length, and interbout interval. A bout was defined as a series of 20 or more licks separated by less than 1 min. Similar to results in an operant oral self-administration procedure described later in this paper (Shabani et al., 2012a), the MADR lines did not differ in MA consumption on the first day that a 20 mg/l MA solution was offered. However, MA consumption diverged after the second day and diverged even more with increases in MA concentration (mice were tested at concentrations of 20, 40, and 80 mg/l in this study). MAHDR mice escalated their MA consumption with each increase of MA concentration, but MALDR consumed negligible amounts. Furthermore, MA blood levels measured after a 2 and 4 h MA drinking session in a 2-bottle choice procedure matched the MA drinking patterns in the MADR lines. Latency to first bout, which can be viewed as an appetitive behavior, was similar at first, but after the first day it diverged in a similar fashion to MA consumption. Shyness to lick the sipper with the MA solution and subsequent lower MA consumption by MALDR mice is an indication of rapid development of MA aversion. All other patterns of consumption behaviors measured via lickometer supported the MA consumption data and the difference between the MAHDR and MALDR lines.

In the sections below, we describe additional research that has been performed in these lines, which provides evidence of their usefulness as a genetic animal model of both genetic risk for and genetic protection from MA use. We also discuss at some length, the potential role of the trace amine-associated receptor 1 (TAAR1) in the difference in MA intake and several other divergent MA-related behavioral traits.

### MA reward and aversion sensitivity in MAHDR and MALDR mice

Important advances in our understanding of the genetic contribution to a number of MA addiction-related traits have been made using our genetic mouse model of differential MA consumption. Robust divergence in voluntary MA intake in the MADR lines has been matched with significant divergence in sensitivity to the rewarding and aversive effects of MA. In two independently selectively bred, replicate sets of MADR lines, MA-induced CPP has been measured using well established methods in which MA was paired with a distinctive tactile cue and then the preference for that cue was later examined, both in the absence and in the presence of MA treatment. MAHDR mice exhibited MA-induced CPP in a drug-free CPP test (saline was administered prior to the test); whereas, no preference for MA-associated cues was seen in MALDR mice, regardless of the conditioning dose (Wheeler et al., 2009; Shabani et al., 2011). The divergence in MA-induced motivational responses between the MADR lines was especially apparent when they were tested in a drug-present CPP test (animals are given the same MA dose they had received during conditioning, immediately before the preference test). The MA dose of 0.5 mg/kg induced robust CPP in MAHDR mice, whereas place aversion was seen in MALDR mice regardless of dose (0.5, 2, and 4 mg/kg). There were no differences in locomotor activity between the MADR lines after treatment with the 0.5 mg/kg MA dose on the drug-present test day; thus, differential activation by MA could not account for their difference in the expression of preference/aversion at this dose. At 2 and 4 mg/kg MA, locomotor responses were higher in MAHDR than MALDR mice; this could reflect a sensitized response to repeated administration of MA, as MAHDR mice exhibited greater sensitization than MALDR mice after repeated administration of MA during the MA conditioning days, although the difference was significant only for the 4 mg/kg dose (Shabani et al., 2011). Activation could have affected expression of a CPP. For ethanol, higher level of locomotor behavior is related to the reduced expression of CPP (Gremel and Cunningham, 2007; Cunningham, 2014), and the apparent magnitude of CPP was somewhat smaller in MAHDR mice for the higher MA doses. When we directly examined this for individual animals on the drug-present test data, we found that higher levels of activity were associated with lower expression of both preference and aversion. Despite this relationship at the level of individual mice, the MALDR mice expressed clear place aversion after treatment with all 3 MA doses, based on group means. It is also possible that unconditioned aversive effects of MA during conditioning and during testing (such as the hypothermia experienced in MALDR, but not MAHDR, mice; Harkness et al., 2015) enhanced the conditioned aversive responses seen in these mice. These data reflect three important points: (1) that both MADR lines are able to discriminate MA-conditioned cues; (2) that responses to conditioned cues are state-dependent; and (3) that significant conditioned aversion was seen in MALDR mice in the drug-present, but not drug-free state.

Differential sensitivity to aversive effects of MA between the MADR lines has been further confirmed in studies designed to be more sensitive at detecting drug-induced aversion. These procedures are referred to as conditioned taste aversion (CTA) and conditioned place aversion (CPA). In both procedures, MA is administered immediately after cue exposure (i.e., a novel taste for CTA and a tactile cue for CPA), rather than before exposure, as in the CPP procedure. It has been suggested that aversion is seen under these conditions, because initial drug effects (e.g., transition from a normal to intoxicated state), in general, are aversive (Hunt and Amit, 1987; Cunningham et al., 2003, 2006); for example, specific physiological responses that are temporally closer to the cue exposure than other more rewarding effects that take longer to manifest. In these studies (Shabani et al., 2012b), MALDR mice exhibited MA-induced CTA at doses as low as 1 mg/kg, whereas no CTA was seen in MAHDR mice up to a dose of 4 mg/kg, the highest dose we have tested. MALDR mice also exhibited CPA at a lower MA dose (2 mg/kg) than MAHDR mice (4 mg/kg). These differences are not likely accounted for by MA pharmacodynamics. MAHDR mice reached higher MA blood levels than MALDR mice at 15 min post 2 mg/kg MA injection, but the MA clearance curves for the two lines were similar (Shabani et al., 2012b). Insensitivity to aversive effects of MA in MAHDR mice appears to be unique to MA, because they did exhibit cocaine-induced CTA (Gubner et al., 2013). The traits described above for which the MADR lines differ, are genetically correlated with MA intake. This suggests some common genetic regulation across these traits, and indicates that at least some of the genetic factors that influence sensitivity to conditioned rewarding and aversive effects of MA also have an impact on MA consumption and seeking (drug-associated cue seeking in the CPP test has been sometimes interpreted as drug seeking). Data for cocaine indicate that learning deficits were not involved in the absence of conditioned responses in either of the lines and that genetic factors influencing these MA-related traits do not influence some similar responses to cocaine. For example, similar levels of both cocaine-induced CPP and CTA were seen in MAHDR and MALDR mice (Gubner et al., 2013) and mice of both lines exhibited equivalent learning in operant procedures (Shabani et al., 2012a). It should be noted that there may be an innate difference between the lines in spatial memory retention, as indicated by a deficit in MAHDR mice in the Morris water maze test (Olsen et al., 2013); however, testing in an additional set of MADR lines is needed to examine replicability of this result and its association with risk for MA consumption.

Studies in the MADR lines strongly support the view that higher MA consumption, which reflects greater vulnerability to continue drug use beyond initial experimentation, is genetically related to greater sensitivity to the rewarding and reduced sensitivity to the aversive effects of the drug. This relationship does not appear to be unique to MA. For example, rat strains with high sensitivity to aversive effects of morphine, ethanol, or nicotine display lower self-administration of those drugs compared to strains with low sensitivity to the aversive effects (Lancellotti et al., 2001; Sánchez-Cardoso et al., 2007; Davis and Riley, 2010). Similarly, rat strains with higher sensitivity to rewarding effects of cocaine (Kosten et al., 1994) acquire cocaine self-administration more rapidly (Kosten et al., 1997). Because these studies utilized only 2 rat strains, definitive conclusions about genetic relationships cannot be drawn. However, studies in larger panels of inbred mouse strains and in selected rat and mouse lines bred for high and low ethanol intake consistently indicate a genetic relationship between ethanol drinking and sensitivity to aversive effects of ethanol (Froehlich et al., 1988; Chester et al., 2003; Green and Grahame, 2008; Cunningham, 2014; Barkley-Levenson et al., 2015), though not in all cases (see Green and Grahame, 2008). Data are less consistent with regard to the relationship between sensitivity to ethanol reward and ethanol intake (Grahame et al., 1999; Phillips et al., 2005; Green and Grahame, 2008; Cunningham, 2014). Some discrepancy may relate to differences in genetic background of the models used in these studies and additional research is needed to examine these relationships for MA in genotypes other than those derived from the B6 and D2 inbred mouse strains.

### MA reinforcement

In addiction disorders, drug consumption or “taking” patterns interact with drug seeking, an appetitive behavior, such that binge-abstinence patterns of drug intake, over time, enhance appetitive behavior (Roberts et al., 2013). However, there are methods that allow consummatory and appetitive behaviors to be considered independently and they appear to be regulated by at least some separate neural circuits. For example, in some operant oral ethanol self-administration procedures, the instrumental behavior, in the form of lever pressing that triggers access to ethanol, is separated from the consummatory behavior, voluntary drinking of the ethanol. Pharmacological manipulations that affect the consummatory behavior do not necessarily affect the appetitive behavior (Czachowski et al., 2002; Ford et al., 2007) and *vice versa* (Czachowski et al., 2002). Evidence indicating independent regulation of appetitive and consummatory behaviors has been generated for psychostimulants as well, though unlike the ethanol studies, operant intravenous self-administration procedures were used (Roberts et al., 2013).

To examine both MA appetitive and consummatory behaviors in our genetic model of differential MA intake, we developed an operant oral MA self-administration procedure (Shabani et al., 2012a). Active lever pressing triggered a light cue above the lever, turned off the house light, and lowered a sipper tube into the chamber for a limited period of time, from which the animal could drink. Pressing of an inactive lever had no consequence. Initially animals were reinforced with a 0.2% saccharin solution on a fixed ratio 1 (FR1) schedule (animals gained access to the saccharin solution each time they pressed the active lever), and then MA was added into the saccharin solution in a stepwise fashion (up to 40 mg/l MA, the highest concentration used during selective breeding). The schedule was then changed to FR2 (animals had to press the lever twice to gain access to the drinking tube) and saccharin was faded completely out of the MA solution in a stepwise fashion, so that it was ultimately presented in water alone (no saccharin). A control group was maintained on the same schedule of reinforcement and animals in that group could press the active lever to obtain access to the same solutions, minus MA. Therefore, the control group was pressing only for water at the end of the study, and provided information about operant behavior in the absence of either saccharin or MA reinforcement. Similar to results for the lickometer study (Eastwood et al., 2014), the two MADR lines did not differ in MA consumption on the first day that MA was added to the solution. However, MA consumption diverged immediately thereafter, with MAHDR mice consuming significantly more MA than MALDR mice. With regard to the appetitive behavior of active lever pressing, there was little difference between the MADR lines when the FR1 schedule was in place; however, MAHDR mice approximately doubled their responding for the MA-containing solution when the demand for MA access was increased by instating an FR2 schedule, whereas MALDR mice retained a lower response rate. The lines of mice did not differ in response rate on the inactive lever, which remained low. These data led to two significant conclusions: (1) pharmacological effects appear to account for divergence in MA consumption in the MADR lines (since the lines consumed similar amounts initially, it is not likely that taste of the MA solution accounted for the subsequent line difference in intake), and (2) genetic risk for increased MA intake is associated with increased appetitive responding for MA; i.e., greater MA reinforcement. We have also confirmed that MAHDR mice will self-administer MA using an operant intracerebroventricular self-administration procedure, which bypasses the oral route, whereas MALDR mice do not acquire this behavior (see Shabani et al., 2012a). Finally, solution intake in the control group declined with saccharin removal. When the schedule was transitioned to FR2, the MALDR, but not MAHDR, control group increased their appetitive responding. This suggests that the MALDR line is more sensitive to the reinforcing effects of natural rewards than the MAHDR line, as they continued to respond for cues associated with the tube that had contained saccharin without MA, even after saccharin had been faded out. Reduced sensitivity to natural rewards could be a potential factor driving higher drug intake in the MAHDR line.

One other procedure that we used to examine reinforcing efficacy was tracking of the reinforced lever after reversal of its location with the non-reinforced lever. MAHDR mice exhibited clear tracking of the MA-reinforced lever and maintained significantly higher MA-reinforced presses on the lever in the new location than on the non-reinforced lever, compared to the MALDR mice. On the other hand, the MALDR control group exhibited stronger tracking of lever reversal than did the MAHDR control group. The lever reversal test measures seeking behavior independent of consummatory behavior, because there is no change in location or mode of reinforcer presentation, and thus, consummatory behavior is minimally affected. In retrospect, to measure MA reinforcement we could also have omitted MA from the solution during the lever reversal procedure. This would have been more like what the control group experienced, since saccharin had been completely faded from the solution, and would provide a test in which seeking behavior is unfettered by postingestive effects of MA. A progressive ratio schedule, in which the behavioral demand is increased over time (i.e., the FR requirement is increased until the animal stops pressing for drug access), is another method that we plan to use in a future study to confirm our findings.

Operant methods have also been used to test the hypothesis that the difference in MA consumption between the MADR lines is related to a difference in impulsive-like behavior. To examine this, a go/no-go task was employed in which mice were trained to perform a nose-poke task to gain access to sucrose, according to specific signaled (cued) requirements (Moschak et al., 2012). Responding at appropriate and inappropriate times was recorded and provided measures of false alarms, hits, and behavioral inhibition (i.e., the ability to withhold responding during the precue period). The MADR lines did not differ in behavioral inhibition when untreated or when treated with MA. In the drug-naïve state, MALDR mice did exhibit a greater hit rate to the visual “go” cue, suggesting that they were more responsive to the cue. Whether salience or visual acuity had a role in this difference is currently unknown; however, a relationship between impulsive-like behavior and genetic susceptibility to MA intake was not found.

Finally, to our knowledge, this is the first genetic animal model specifically created for a trait related to MA intake. However, lines of rats bred for high (HiS) and low (LoS) saccharin intake deserve mention (Dess and Minor, 1996), based on evidence of the involvement of natural reward pathways in the effects of drugs of abuse (Joffe et al., 2014) and of predictability of level of AMPH self-administration from individual differences in sucrose intake (DeSousa et al., 2000). Compared to the LoS line, the HiS line exhibits a higher level of operant responding for sucrose (Gosnell et al., 2010), consumes more ethanol (Dess et al., 1998), self-administers more cocaine (Morgan et al., 2005), and shows more rapid escalation of cocaine intake (Perry et al., 2006). These data support a link between avidity for a natural reward and for several drugs of abuse, but to our knowledge, these lines have not been studied for their avidity for AMPH or MA. Our operant data in the MADR lines suggest the opposite relationship between avidity for saccharin and MA (Shabani et al., 2012a).

### Physiological responses to MA

MA is a powerful sympathomimetic with dose-dependent effects on body temperature, heart rate, and blood pressure, and thus, it disrupts homeostasis (Rusyniak et al., 2012; Harkness et al., 2015). MA may produce its effects on body temperature through both central nervous system and peripheral targets (Matsumoto et al., 2014). Our recent data reveal a highly significant divergence in MA-induced thermic response between MAHDR and MALDR mice (Harkness et al., 2015). Lower doses of acutely administered MA (1–4 mg/kg) induced hypothermia in MALDR mice, an effect not seen in MAHDR mice at any dose. Doses of MA from 1 to 16 mg/kg induced hyperthermia in MAHDR mice, whereas hyperthermia was seen in one replicate line of MALDR mice only after treatment with 16 mg/kg MA. Our previous published work indicates no difference in initial MA-induced locomotor response between the two lines (Shabani et al., 2011); therefore these differences in MA-induced thermic effects are not likely accounted for by differences in overall locomotor activity. Furthermore, animals are individually housed in small chambers that restrict locomotor behavior. These chambers are used to prevent changes in body temperature via heat exchange between cagemates. Overall, these data indicate that MA-induced hypothermia is associated with lower MA intake and may be a protective physiological response. On the other hand, greater sensitivity to the hyperthermic response to MA does not appear to protect against MA consumption, and instead was associated with greater risk for MA consumption.

Differential MA-induced activation of the sympathetic nervous system could play a role in the difference between the MADR lines in thermal response to MA. In a recent study in anesthetized rats, MA infused into the medial prefrontal cortex (mPFC) evoked interscapular brown adipose tissue thermogenesis, a sympathetic nervous system-mediated effect (Hassan et al., 2015). Furthermore, norepinephrine, but not DA, infused into the mPFC mimicked the MA effect. MA increased extracellular DA in the mPFC of MAHDR, but not MALDR, mice (Lominac et al., 2014), whereas MA had no effect on extracellular serotonin in the mPFC of MAHDR mice, but increased extracellular serotonin in the mPFC of MALDR mice. Neither of these effects of MA was seen in the nucleus accumbens (NAc). MA-induced extracellular norepinephrine levels have not been measured in response to MA in these mice. Rats with their adrenal glands removed failed to exhibit a hyperthermic response to MA, but dexamethasone, a corticosterone-like drug, “rescued” this MA-induced hyperthermic response (Makisumi et al., 1998). Furthermore, sympathetic blockade via peripheral depletion of catecholamines abolished the MA-induced hyperthermic effect. Our mouse model provides a unique opportunity to determine mechanisms of MA-induced thermic effects in the central nervous system, as well as the peripheral nervous system and its targets.

Because the MA-induced thermic responses occur rapidly after injection, it is reasonable to assume that animals make strong associations between the unconditioned physiological effect and the unique cues used in conditioning studies. Therefore, these physiological effects of MA could have a role in the conditioned responses. For example, it is possible that different behavioral outcomes are seen in the drug-present and drug-free preference tests, in part, because MA-induced physiological cues are missing during the drug-free CPP test. Harkness et al. (2015) explored thermic responses to a single acute bolus MA administration, whereas the behavioral response in the drug-present CPP test was examined after repeated MA administrations. Repeated MA administration in a rat model did not diminish hyperthermic responses under similar conditions to ours (Myles et al., 2008). However, hypothermic responses were enhanced. In addition, MA-induced thermic effects can be dependent on ambient temperature (Myles et al., 2008; Sabol et al., 2013), which we have not yet explored, nor have we explored other MA-induced sympathomimetic effects, such as heart rate, breathing rate, and blood pressure, which could also affect thermic responses and motivated behaviors.

Finally, another effect of MA that could impact risk for its use, is its circadian rhythm disrupting effect (e.g., Pendergast et al., 2013; Pendergast and Yamazaki, 2014). We examined the free-running circadian period of the MADR lines and found no difference in drug-naïve mice, but greater sensitivity in the MALDR line to the effect of voluntarily consumed MA to lengthen the circadian period. The MALDR line was significantly more sensitive to this MA effect, although they consumed significantly less MA than the MAHDR line. These data suggest that MA effects on circadian rhythm may also be protective against MA consumption (Olsen et al., 2013). We have not examined whether circadian rhythm disturbance might alter MA intake, but a recent study by Doyle et al. (2015) reported that MA consumption was increased in phase-shifted rats that had a history of MA use.

### Quantitative trait locus and expression analyses

The heritability of two-bottle choice MA consumption in the replicate 1 and 2 lines was ~0.35 (Wheeler et al., 2009; Shabani et al., 2011), indicating that 35% of the variance in MA intake can be attributed to genetic differences. In an initial study, we examined the potential involvement of a select set of genes (384 chosen for their potential involvement in psychiatric disorders, including addiction) by studying their expression levels in NAc tissue from MA-naïve and MA-treated MAHDR and MALDR mice. A quantitative PCR gene array (Mouse Mood Disorder StellARray) was used. In MA-naïve mice, there were multiple genes that were different in basal level of expression between the selected lines, including serotonin and noradrenaline transporter genes (but not DA transporter) and a metabotropic glutamate receptor gene, as well as genes for adenylate cyclase activating polypeptide 1, glutathione peroxidase 1, and others too numerous to mention. Detailed results are presented in Wheeler et al. (2009). In mice given an acute injection of 2 mg/kg MA, a dose that induces different reward- and aversion-related traits in the MAHDR and MALDR lines, MA-induced changes in gene expression were largely line-specific, but it is worth noting that MA appeared to have the effect of altering expression to counteract differences in basal expression level that were seen between the lines.

Genes involved in apoptosis, immune response, and cytokine signaling were enriched among those that were regulated by MA in the MALDR line, whereas genes relevant to toll-like receptor signaling were regulated by MA in the MAHDR line. Given recent data suggesting that neuroimmune signaling plays a role in biological processes associated with addiction (Cui et al., 2014; Ray et al., 2014), studies are currently examining neuroimmune responses to acute and repeated MA treatment at the level of specific cytokines and chemokines to determine their potential involvement in the difference in MA intake between the MAHDR and MALDR lines.

To identify the chromosomal locations of influential genes, QTL analysis was conducted (Belknap et al., 2013). QTL mapping results were reproducible and indicated a large effect QTL on chromosome 10 that accounts for more than 50% of the genetic variance in MA consumption. There were also several genome-wide significant QTLs on other chromosomes, which had smaller magnitude effects than the chromosome 10 QTL. Also described in this published paper are the results of microarray gene expression (Affymetrix) analyses in MA-naïve MAHDR and MALDR mice for 3 brain regions, the NAc, prefrontal cortex, and ventral midbrain (including the substantia nigra and ventral tegmental area). In the chromosome 10 QTL location, there was similar clustering of differentially expressed probe sets for the 3 brain regions, although the specific probe sets making up those clusters differed among the brain regions. A comparative network analysis was performed to identify groups of differentially expressed genes with coordinated gene function, and found a significant transcription factor rich subnetwork that was functionally enriched for the regulation of apoptotic processes that could have a role in neuroimmune signaling. This supported further study of neuroimmune signaling involvement in not only the effects of MA, but also in risk for MA use.

### Behavioral MA-related traits in the progenitor strains of the MADR lines

The B6 and D2 progenitor strain mice have been characterized for some MA-related traits that are similar to those examined in the MADR lines. D2 mice consumed more MA than B6 mice (Eastwood and Phillips, 2012), which is consistent with the association of D2 alleles on chromosome 10 with high MA intake (Belknap et al., 2013). Separate studies with B6 and D2 mice have reported sensitivity to MA-induced CPP in both strains (Cunningham and Noble, 1992; Thanos et al., 2010; Bryant et al., 2012; Dobbs and Cunningham, 2014; Lominac et al., 2014), and we have been unable to find a study that directly compared these strains. Testing these strains in a CPP study identical to ours would be important for determining whether a progenitor strain difference exists that is comparable to our MADR line difference. In that case, we would expect D2 mice to exhibit greater sensitivity to MA-induced CPP, compared to B6 mice. With regard to MA effects on body temperature, D2 mice exhibited an AMPH-induced hyperthermic response, whereas B6 mice exhibited greater hypothermia, and blunted MA-induced hyperthermia (Seale et al., 1985), traits that are consistent with the relationships found in the MADR lines. Recombinant inbred strains were tested to determine whether the differential high dose AMPH-induced hyperthermic response had a polygenic influence. In fact, only two AMPH-induced thermic response phenotypes were found, rather than a continuous distribution, supporting the influence of a single gene. Results in another study using D2 and B6 mice indicated little difference between the strains. However, temperature was assessed at only a single time point in that study (Grisel et al., 1997).

With regard to locomotor responses to MA or AMPH, in two studies, somewhat larger initial stimulant responses, depending on dose, were seen in B6, compared to D2 mice (Phillips et al., 1994; McNamara et al., 2006). However, the Phillips et al. (1994) study revealed a greater magnitude of sensitization to 1 and 2 mg/kg MA in D2, compared to B6, mice (Phillips et al., 1994). In another study, B6 mice did not exhibit significant AMPH-induced locomotor activation at any dose, whereas significant increases in locomotor behavior in response to some AMPH doses, but locomotor depression in response to others was found in D2 mice (Anisman et al., 1975). D2 and B6 strains from a different vendor (i.e., Charles River, Italy) both exhibited significant locomotor activation to 2.5 mg/kg AMPH, but the AMPH-induced locomotor response was lower in D2 than B6 mice (Zocchi et al., 1998; Ventura et al., 2004). Our data for the MADR lines have not indicated any consistent differences in sensitivity to the acute stimulant effects of MA. Furthermore, gene mapping in lines of mice bred from the B6D2F2 for differential sensitivity to the acute stimulant effects of MA did not identify a QTL on chromosome 10 (Kamens et al., 2005; Palmer et al., 2005), nor did mapping in lines of mice bred from the B6D2F2 for differential magnitude of MA-induced sensitization (Scibelli et al., 2011; Belknap et al., 2013). Therefore, if there are differences between the MADR lines and their progenitor strains in locomotor activation by MA, they may be unrelated to polymorphic genes associated with the large effect QTL for MA intake on chromosome 10.

### Candidate gene and mechanism studies

#### The mu-opioid receptor gene, *Oprm1*

The chromosome 10 QTL has not yet been fine-mapped and the interval includes a large number of genes. Included among them is *Oprm1*, for which our microarray analysis identified greater expression in prefrontal cortex tissue from MALDR, compared to MAHDR, mice. D2 alleles are found at higher frequency in MAHDR mice than MALDR mice in the chromosome 10 QTL region and thus, are associated with higher MA intake (Belknap et al., 2013). Furthermore, D2 and B6 mice have been found to differ in several opioid-related responses, including sensitivity to the acute stimulant effects of morphine, sensitivity to morphine-induced analgesia, and morphine intake (Belknap et al., 1989, 1993; Berrettini et al., 1994a,b; Phillips et al., 1994; Bergeson et al., 2001). We therefore performed a series of studies designed to ascertain whether *Oprm1* is a viable candidate as an influential quantitative trait gene in our QTL region. MALDR mice were found to be more sensitive than MAHDR mice to the locomotor stimulating, but not analgesic, effects of mu opioid receptor (MOP-r) agonist drugs (Eastwood and Phillips, 2012). In addition, MALDR mice voluntarily consumed higher amounts of morphine than MAHDR mice, and MOP-r agonists, but not antagonists, reduced MA intake in MAHDR mice (Eastwood and Phillips, 2014). Furthermore, we found higher MA intake in D2 than in B6 mice, consistent with the largely D2 genotype of MAHDR mice on chromosome 10 (Eastwood and Phillips, 2012). These findings support genetic correlations between some opioid drug responses and MA intake and thus, suggest common genetic regulation of these traits. However, linkage as an explanation for these results cannot currently be ruled out. The D2-like *Oprm1* allele may occur at higher frequency in the MAHDR line because it is linked to the gene that influences MA intake, resulting in these line-specific opioid responses. In fact, our network analysis did not identify *Oprm1* as a member of the gene network regulating MA consumption, but when it was added to the network, it exhibited regulation by several transcription factor genes in the network (Belknap et al., 2013).

#### The trace amine-associated receptor 1 gene, *Taar1*

Another gene within the chromosome 10 QTL confidence interval is *Taar1*. Our recent data strongly suggest that it has a significant role in MA drinking (Harkness et al., 2015). Our interest in this gene was stimulated by information in existing databases that indicated that the D2 progenitor of the MADR lines possesses a non-synonymous mutation in *Taar1*, which we subsequently determined codes for a non-functional receptor (TAAR1). This polymorphism is unique to the D2 strain. A paper published in 2010 (Vallender et al., 2010) detailed the functional evolution of TAAR1 and concluded that *Taar1* appears to be under strong purifying selection across mammals, avians, and amphibians. However, in the dog, *Taar1* has become a pseudogene, and in D2 mice, this non-synonymous mutation has ablated the production of a functional receptor. It is of great interest to us that a study in dogs found them to respond to oral doses of d-AMPH (0.25, 0.75, 1.5 mg/kg) with only hyperthermia (Tontodonati et al., 2007). This is a trait we see in MAHDR mice that we believe is linked to TAAR1 non-functionality, based on information given below and in Harkness et al. (2015).

TAAR receptors belong to the family of G-protein coupled receptors, but only TAAR1 and perhaps TAAR4 exhibit high sensitivity to trace amines and AMPH-like compounds (Borowsky et al., 2001; Bunzow et al., 2001). These endogenous amine compounds are found in trace amounts, often, but not always, in catecholaminergic systems of the mammalian nervous system. Trace amines are viewed as intimate endogenous neuromodulators of monoaminergic neurotransmission, although both trace amines and AMPH-like compounds can act on targets other than TAAR receptors (for review see Berry, 2004). When 10 MAHDR and 10 MALDR mice were sequenced at the location of the D2 polymorphism, MAHDR mice were found to be homozygous for the non-functional D2 *Taar1* allele, whereas MALDR were either homozygous for the alternative functional B6 *Taar1* allele or heterozygous (Harkness et al., 2015). Thus, there is an association of the *Taar1* polymorphism with MA intake, and perhaps other MA-related traits that differentiate the MAHDR and MALDR lines.

To further examine this relationship, *Taar1* transgenic mice were tested for MA consumption. *Taar1* knockout mice, which are homozygous for a null mutant *Taar1* allele, and thus, lack functional TAAR1, exhibited significantly higher levels of MA intake, compared to *Taar1* heterozygote or wildtype mice. In addition, we found that functional TAAR1 increases sensitivity to hypothermic effects of MA and to MA-induced CTA, as indicated by higher levels of these traits in *Taar1* heterozygote and wildtype mice, compared to knockout mice. However, neither the MADR lines, nor the transgenic genotypes, differed in sensitivity to the hypothermic effects of ethanol (Harkness et al., 2015). Previous data from *Taar1* transgenic mice indicate altered monoaminergic systems. For example, ventral tegmental area clamp recordings (Lindemann et al., 2008) of DA neurons from *Taar1* knockout mice revealed significantly higher spontaneous spike frequency and a more depolarized resting membrane potential, compared to recordings for wildtype mice. The mean spike frequency of DA neurons from wildtype mice was suppressed by p-tyramine, a TAAR1 agonist, an effect that was absent in *Taar1* knockout mice. Despite these differences in firing frequencies of DA cells, the extracellular DA and norepinephrine levels in the striatum were the same in wildtype and *Taar1* knockout mice (Lindemann et al., 2008). Lindemann et al. (2008) suggested that higher DA neuronal activity in *Taar1* knockout mice does not lead to higher extracellular DA release because lack of TAAR1 activity reduces efflux and inhibition of DA reuptake; *in vitro* studies support this view (Miller, 2011). Monoamine reuptake transporters serve as conduits to TAAR1 substrates, including AMPH or MA, and in turn, signals from activation of TAAR1 inhibit monoamine uptake transporters. Furthermore, MA can reduce cell surface monoamine uptake transporters by as much as 70% in a TAAR1-dependent manner (Xie and Miller, 2009). AMPH induces significantly greater increases in extracellular DA and norepinephrine in *Taar1* knockout than wildtype mice (Wolinsky et al., 2007; Lindemann et al., 2008). One hypothesis is that lack of TAAR1 results in less internalization of monoamine uptake transporters and thus, greater monoamine uptake inhibition by AMPH or MA. In *Taar1* knockout mice, lack of tonic inhibition by TAAR1, which translates into higher catecholaminergic activity, can lead to quick increases in extracellular DA if uptake transporters are blocked by AMPH or MA (Miller, 2011). Although there has been significant recent research progress on the function of TAAR1, it is still at a nascent stage in connecting cellular mechanisms to behavioral effects, but see Miller (2011), Lindemann and Hoener (2005), and Berry (2004).

Most of our recent data in *Taar1* transgenic mice indicate that the functional *Taar1* allele is dominant for low MA consumption, sensitivity to the aversive effect of MA, and MA-induced hypothermia (Harkness et al., 2015). Thus, mice homozygous for the *Taar1* mutant allele, exhibited a difference in phenotype, compared to both heterozygous and homozygous mice, which have at least one copy of the allele that produces a functional TAAR1. We have not yet determined how heterozygous mice differ from homozygous wildtype mice, with regard to neurochemical traits relevant to TAAR1 function, and we do not know whether or not twice as many functional receptors are expressed by the wildtype vs. heterozygous mice. If that is the case, then our data suggest that a reduction in receptor number, even by half, does not impact these traits, and TAAR1 continues to play a protective role.

The fold difference in MA intake between MAHDR and MALDR mice is larger than in the progenitor strains (B6 and D2) and the *Taar1* knockout vs. wildtype mice (Harkness et al., 2015). This could reflect the impact of genes other than *Taar1* on MA drinking that were affected by selective breeding and would be uniquely different in the oppositely selected MADR lines. Mapping results identified additional locations of QTL for MA drinking that had effects of smaller magnitude, compared to the QTL on chromosome 10 (Belknap et al., 2013). However, since the chromosome 10 QTL accounts for only approximately half of the genetic variance in MA consumption, genes at other locations are of interest, as are gene interactions (networks) and the possibility that there are genes that act as “hubs.” Hubs are genes that are highly interconnected with other genes in a network and studies designed to manipulate such hubs and look at phenotypic impacts are planned.

Studies in rats have examined the effects of TAAR1 agonists on operant MA self-administration. The TAAR1 agonists, RO5203648 and RO5263397, significantly reduced the number of MA infusions self-administered (Cotter et al., 2015; Jing et al., 2015). Although studies have not been done to determine whether these agonists produce their effects by reducing the reinforcing effects or increasing the aversive effects of MA, our data suggest that the latter is more likely. This approach to reduce MA consumption in the MADR mice is not possible, because the TAAR1 in those mice is completely non-functional and an increase in function would not be afforded by agonist treatment. We are currently exploring human *Taar1* polymorphisms to determine what the range of TAAR1 function is in human populations, and thus, whether the development of TAAR1 agonists is a viable consideration for MA addiction treatment.

TAAR1 appears to be essential for MA-induced hypothermic effects. MA and AMPH are potent agonists for TAAR1 and TAAR1 is highly expressed in brain areas known for thermoregulation, such as the preoptic area (Lindemann et al., 2008; Morrison and Nakamura, 2011). A 20 mg/kg dose of MA dramatically increased cFos expression in the medial preoptic area of B6 mice (Tomita et al., 2013). However, TAAR1 is also expressed peripherally, and data do not yet exist that have teased apart whether MA mediates thermic responses through peripheral targets, the central nervous system or both (Broadley, 2010; Panas et al., 2012). In contrast to the results of Harkness et al. (2015), a recent study in independently created *Taar1* transgenic mice found a similar hyperthermic response to a 3 mg/kg dose of MA in wildtype and *Taar1* knockout mice (Panas et al., 2010). However, the investigators did not test other doses, such as a dose that induced hypothermia in their wildtype mice, so it is not clear if these results are actually disparate from ours. In addition, the method of temperature measurement in the Harkness et al. (2015) study was via rectal probe, whereas that in Panas et al. (2010) was via a wireless infrared thermometer over the shaved lower back area of the mouse.

We have not examined MA-induced locomotor activation or sensitization in our *Taar1* transgenic mice; however, some such studies exist in independently generated lines. Most of the studies that have examined MA or AMPH effects on locomotor activity, indicate that wildtype and *Taar1* knockout mice are not different in baseline locomotor activity, but do differ in stimulant response, although the data have not been entirely consistent with regard to dose-dependent differences. Thus, *Taar1* knockout mice exhibited higher locomotor activation to acute 1 and 2.5 mg/kg AMPH than wildtype mice in a study by Lindemann et al. (2008), whereas Achat-Mendes et al. (2012) observed similar locomotor responses to 1 and 5 mg/kg AMPH, but greater activation in *Taar1* knockout mice treated with 3 mg/kg AMPH or MA, compared to wildtype mice. Wolinsky et al. (2007) found greater activation in *Taar1* knockout mice in response to 1 mg/kg AMPH, but not 2.5 or 5 mg/kg. But overall, *Taar1* knockout mice have exhibited greater sensitivity to AMPH-like drug stimulation. The variability in dose responses could be related to differences in the genetic background of the various lines. For example, both the C57BL/6J and C57BL/6N have been used in the generation of various knockout lines. These two strains are separated by some 220 generations; thus, their genomes have a number of variations in their coding sequences that could influence any number of phenotypes (Simon et al., 2013). Furthermore, spontaneous and naturally occurring mutations in coding regions have occurred in strains maintained by specific breeding vendors, as, for example, for the exon deletion from *Klrd1* in D2 mice coming from The Jackson Laboratory, but not from other vendors (Shin et al., 2015). In fact, we are currently collecting sequence data from recombinant inbred strains derived from D2 and B6 progenitors at different times in history that suggest the *Taar1* non-functional allele may be a newer spontaneously occurring mutation in the D2 strain.

## Conclusions and future directions

MADR mice provide a unique opportunity to further explore the contribution of *Taar1* and TAAR1 to voluntary MA consumption, and to the acute and repeated MA-related effects that are genetically correlated with consumption and also seem to be regulated by this gene and its receptor. Decades of research on genetic risk for alcoholism has led to the conclusion that rare single gene variants with large impacts might still be found, but that common variants in multiple genes, each with a small effect, are those more likely to be implicated (Enoch, 2013). Less is known about the genetic architecture of risk for MA abuse, and the large effect QTL found in our selected lines could represent the impact of a rare variant. Genetic investigation in humans is needed to address this question. However, a gene or genes in the chromosome 10 QTL region accounts for about half of the genetic variance in the MA consumption trait, thus, additional research is needed to examine the impact of other genes and how they interact to increase risk for MA use. We are currently developing a binge model of MA intake using the MAHDR line and studying how differing periods of withdrawal from chronic MA use might impact their drinking behavior. These and other studies are intended to improve our genetic model with regard to its translational value to human addiction. In this way, important mechanisms may be identified, with druggable targets, for the development of pharmacotherapeutics for MA abuse. Another important future direction is to determine the impact of the *Taar1* mutation on MA-related traits on different genetic backgrounds and to study whether there are epistatic (gene × gene) or gene × environment interactions that impact the effect of the *Taar1* mutation.

## Author contributions

TP wrote the abstract and drafted the outline for this review, wrote several sections, revised all sections interactively with SS, and engaged coordinately in final approval and agreement to be accountable for the statements in the paper. SS wrote several sections, participated in revision of the entire paper, and engaged coordinately in final approval and agreement to be accountable for the statements in the paper.

### Conflict of interest statement

The authors declare that the research was conducted in the absence of any commercial or financial relationships that could be construed as a potential conflict of interest. They or their institution did not receive services from a third party for any aspect of the submitted work. They have no financial relationships with entities that could be perceived to influence, or give the appearance of potentially influencing, statements in this paper.
